# The gut-skin axis in wound healing: a bibliometric analysis of intestinal flora and chronic wounds (2005-2025)

**DOI:** 10.3389/fcimb.2026.1800070

**Published:** 2026-04-30

**Authors:** Jing Chen, Aode Tian, Jianliang Huang, Zhenghe Wang, Hongjin Wang

**Affiliations:** 1 Qinghai University, Xining, Qinghai, China; 2 Department of Burns and Plastic Surgery, Qinghai University Affiliated Hospital, Xining, Qinghai, China

**Keywords:** bibliometrics, chronic wound, Citespace, microbiota, refractory wound, the gut-skin axis, VOSviewer

## Abstract

**Background:**

The delayed healing of chronic wounds is a major challenge in clinical treatment, with complex pathogenesis involving multisystem dysfunction. In recent years, the role of intestinal flora imbalance in the impairment of chronic wound healing has received increasing attention. Given the physiological similarities and pathological correlations between the skin and the intestine, some scholars have proposed the concept of the “skin-gut axis” and believe that there is a close connection between the skin microbiota, skin condition, and intestinal flora. However, systematic analysis of research trends remains limited. This bibliometric study comprehensively evaluates the research status in the fields of chronic refractory wounds and intestinal microbiota from 2005 to 2025.

**Methods:**

We used CiteSpace, VOSviewer, and Bibliometrix software to analyze 553 English articles from the Web of Science Core Collection. Bibliometric assessments included publication trends, collaborative networks, geographical distribution, journal impact, and keyword clustering.

**Results:**

A total of 553 papers were included. The number of publications in this field increased markedly from 2020 to 2025, with 2025 being the most productive year. The leading contributing countries were the United States and China; key institutions included the University of Pennsylvania and the University of Copenhagen. Elizabeth A. Grice was the most impactful author. Keyword analysis identified high-frequency terms: wound healing, biofilm, chronic wound, bacteria, and microbiota. Timeline visualization of keywords revealed emerging research hotspots, including diabetic foot ulcer and *in situ* detection.

**Conclusion:**

To the best of our knowledge, this study represents the first bibliometric analysis in the field of chronic wounds and intestinal microbiota. Core research foci include disease-associated microbiota characteristics, probiotic therapy, microecological intervention, and the distal gut–target organ axis. Future research should prioritize microbe-targeted therapy, regulation of the immune–barrier metabolic network, and integrative medicine combining traditional Chinese and Western medicine.

**Systematic Review Registration:**

https://www.crd.york.ac.uk/PROSPERO/ID=CRD420261362660, identifier CRD420261362660.

## 1 Introduction

Chronic wounds are defined as long-standing non-healing wounds that fail to undergo timely and orderly tissue repair following cutaneous injury. Clinically, wounds that do not heal after more than 4 weeks of standard care due to diverse etiologies are classified as chronic wounds. (Frykberg et al., 2015). Chronic wounds represent a substantial and growing global clinical burden, associated with long-term disability, impaired quality of life, substantial economic costs, and elevated risks of secondary complications including surgical site complications (SSC) and surgical site infections (SSI). In 2022, global spending on wound care amounted to US$148.65 billion (Sen, 2025). In the United States, approximately 10.5 million Medicare beneficiaries are affected by chronic wounds, with an annual economic burden of approximately US$22.5 billion (Sen, 2025); worldwide, around 6.7 million individuals live with chronic wounds, imposing a heavy socioeconomic toll (Järbrink et al., 2016). The pathophysiological hallmarks of chronic wounds encompass persistent dysregulated inflammation, impaired angiogenesis, and disrupted extracellular matrix (ECM) remodeling, all of which collectively impede effective tissue repair. Emerging evidence indicates that aberrant macrophage polarization (sustained activation of the pro-inflammatory M1 phenotype) and overactivation of the Toll-like receptor 4 (TLR4)/nuclear factor-κB *NF* – *κB* signaling pathway are central mechanisms underlying prolonged inflammation (Murray et al., 2014); downregulated expression of vascular endothelial growth factor (VEGF) and dysregulated microRNA-21 (miRNA-21) are linked to defective angiogenesis; and an imbalance between matrix metalloproteinases (MMPs) and tissue inhibitors of metalloproteinases (TIMPs) results in disordered collagen deposition (Eming et al., 2007). Common comorbidities include diabetes mellitus, peripheral arterial disease (PAD), and pressure ulcers, all of which compromise healing and increase recurrence risk. By 2025, an estimated 589 million adults globally will be living with diabetes mellitus, who carry a markedly elevated risk of chronic wounds due to concurrent neuropathy and microvascular injury (Duncan et al., 2025). PAD is highly prevalent among patients with diabetes, and their coexistence further increases the risk of adverse limb outcomes such as amputation (Das et al., 2025). In China, the prevalence of diabetic foot ulcers among individuals with diabetes is approximately 6.4%, with more than 1 million new cases annually and 10%–20% of patients at risk of amputation (Chinese Medical Association, 2025); globally, diabetic foot ulcers affect nearly 18.6 million people each year, resulting in over 1.6 million amputations annually (Armstrong et al., 2023).

“The Gut-Skin Axis” and Wound Healing: The skin and intestine share numerous common characteristics. Both intestinal and cutaneous cells originate from the same germ layer, and their surfaces are covered by epithelial cells that are in direct contact with the external environment. These epithelial cells continuously communicate with the external environment and serve as the first line of defense for the human body. Additionally, both the intestine and skin are highly vascularized and highly responsive to nervous system activity; the skin microbiota and intestinal microbiota are the two most important microbial communities in the human body (Byrd et al., 2018). Meanwhile, the skin and intestine are key components of the immune and neuroendocrine systems. There is also evidence of a bidirectional crosstalk between the intestine and skin, which is influenced by diet, mood, and various external factors (Salem et al., 2018). This connection between the skin and intestine can be mediated by the host’s immune system, and the interaction between the immune system and microorganisms is critical for maintaining skin homeostasis (Slominski et al., 2025). Based on these observations, the concept of the “gut-skin axis” has been proposed in recent years, which holds that the interactions among the immune system, neuroendocrine system, and microorganisms are essential for sustaining skin homeostasis.

## 2 Materials and methods

### 2.1 Data source

**Figure 1:**
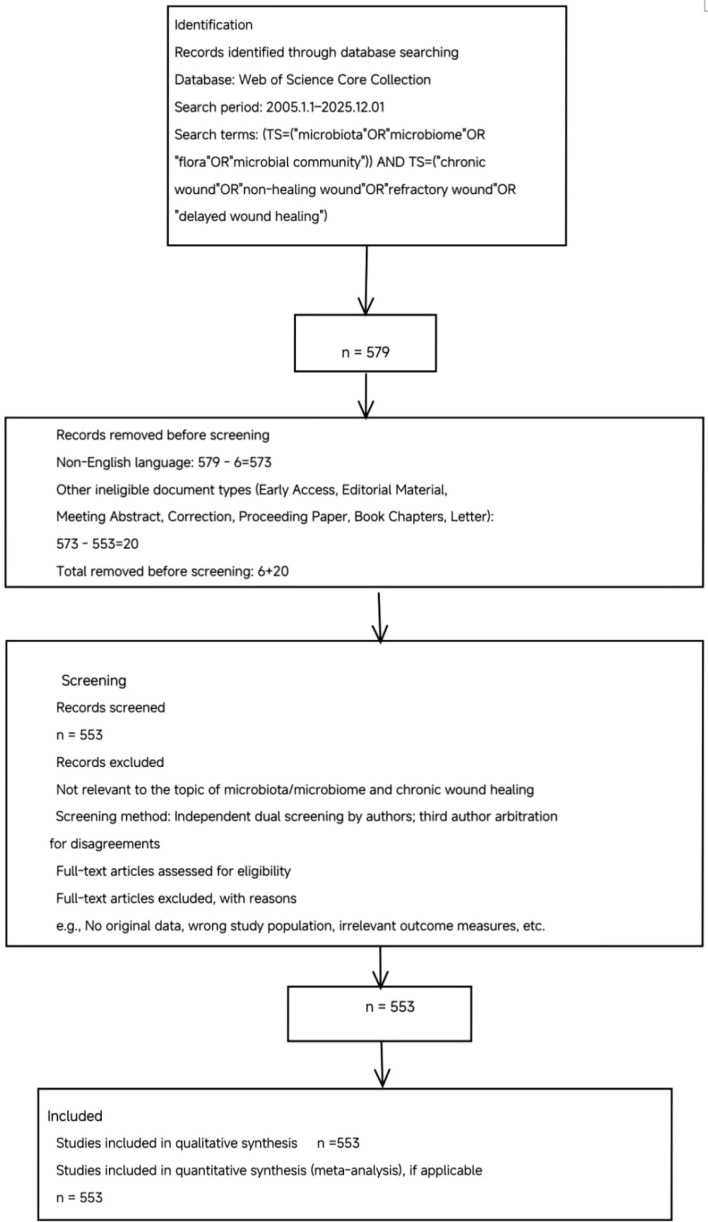
PRISMA flowchart of literature screening for intestinal microbiota and chronic refractory wounds. This flowchart details the systematic literature selection: initial search yielded 579 records, with final exclusion resulting in 553 English-language studies for analysis.

This study followed the PRISMA 2020 guidelines (Page et al., 2021), with a standardized PRISMA flow diagram documenting the study selection process (**Figure 1**). A systematic search was performed in the Web of Science Core Collection (January 1, 2005–December 1, 2025) using the strategy: TS=(“microbiota” or “microbiome” or “flora” or “microbial community”) AND TS=(“chronic wound” or “non-healing wound” or “refractory wound” or “delayed wound healing”), including only English publications.

Studies were included/excluded per pre-set criteria: included original research (randomized controlled trials [RCTs], cohort, cross-sectional studies) focusing on microbiota/microbiome and chronic wound relationships;

Two authors independently screened titles/abstracts and full texts; discrepancies were resolved by a third senior author. Data were extracted using a standardized form, and quality was assessed via the Newcastle-Ottawa Scale (NOS) for observational studies and Cochrane Risk of Bias tool for RCTs.

### 2.2 Data collection and cleaning

A total of 579 articles were initially retrieved and excluded based on pre-defined criteria: non-original/summary articles, withdrawn/duplicate articles, non-English articles, and other non-representative items. After screening, 553 English articles were finally included for analysis (**Figure 1**). Documents were exported as “complete documents with cited references” and “plain text”; all data were extracted on December 1, 2025, to avoid bias from daily database updates.

Extracted information included publication volume, citation frequency, and core details (countries/regions, institutions, authors, references, journals, keywords). All data were obtained from open-access databases following standard protocols. To improve reliability and minimize inaccuracies, potential biases (e.g., inconsistent abbreviations, varied citation versions) were addressed via multi-researcher review, synonym merging, and spelling correction.

### 2.3 Bibliometric analysis

CiteSpace and VOSviewer are two important and commonly used tools in bibliometric analysis. CiteSpace is the most widely utilized bibliometric analysis software, primarily employed for the analysis of bibliometric network diagrams (Chen et al., 2004). In this study, visual analyses were performed on publishing countries, institutions, authors, journals, and keywords. Visualization charts were constructed to summarize the current research progress, hotspots, and emerging trends in the field of intestinal microbiota and chronic wounds. For the CiteSpace software, the analysis time slice was set from January 2005 to December 2025, with a 1-year time interval. The node selection method adopted was the G-index, with the k-value set to 25 (Chen et al., 2017). Keywords were selected using the default settings, and the threshold was maintained at the system default value.

## 3 Result

### 3.1 Analysis of articles published in the year

A total of 553 articles were retrieved between January 1, 2005, and December 1, 2025. In terms of publication volume, the cumulative output increased steadily, reflecting the continuous accumulation of research in this field. Regarding citation metrics, the total number of citations reached 20,738, with 19,160 citations after excluding self-citations, resulting in an average of 37.5 citations per article—indicating high academic influence of the research findings. The H-index was 76, which also intuitively reflects the core value and weight of the research achievements in this field.

**Figure 2:**
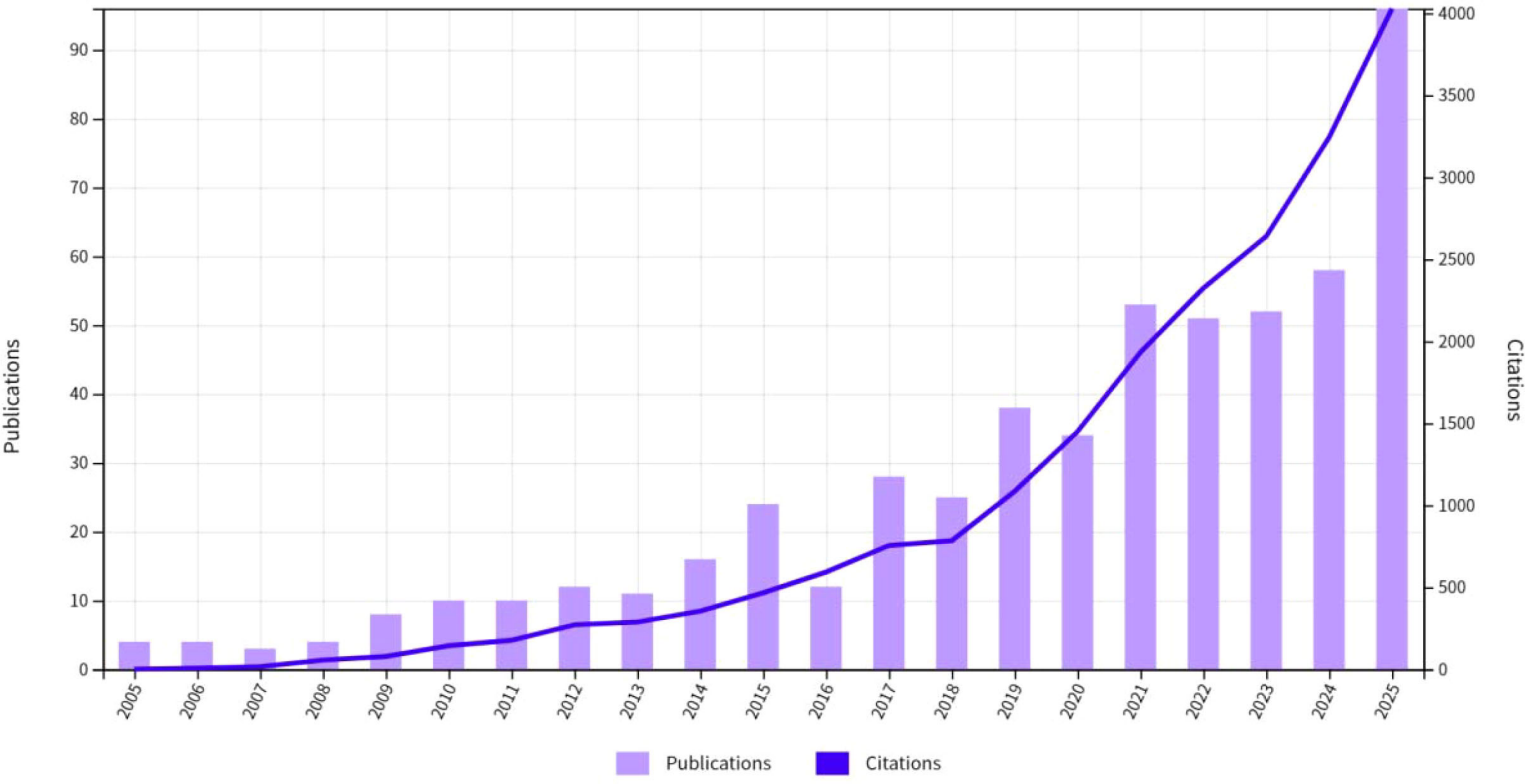
Annual publications and citations from 2005 to 2025. Bars (purple, left y-axis) show annual publication counts, while the line (dark blue, right y-axis) represents corresponding citations. Both metrics grew gradually until 2018, then accelerated sharply, with publications peaking at ~95 and citations exceeding 4000 by 2025, indicating rapidly expanding research output and impact.

Research trends in this field from 2005 to 2025 are illustrated in **Figure 2**, which presents annual publication and citation data. In terms of publication volume, there was a year-on-year increasing trend, particularly after 2020, indicating sustained global attention from researchers in this field. In terms of citation frequency, the total number of citations increased in parallel with the growing publication volume, with a significant surge in citations during 2024–2025. This suggests that the dissemination and influence of research findings have increased rapidly in recent years, and the field has entered an accelerated growth stage of attention.

### 3.2 Countries/regions and institutions

**Figure 3:**
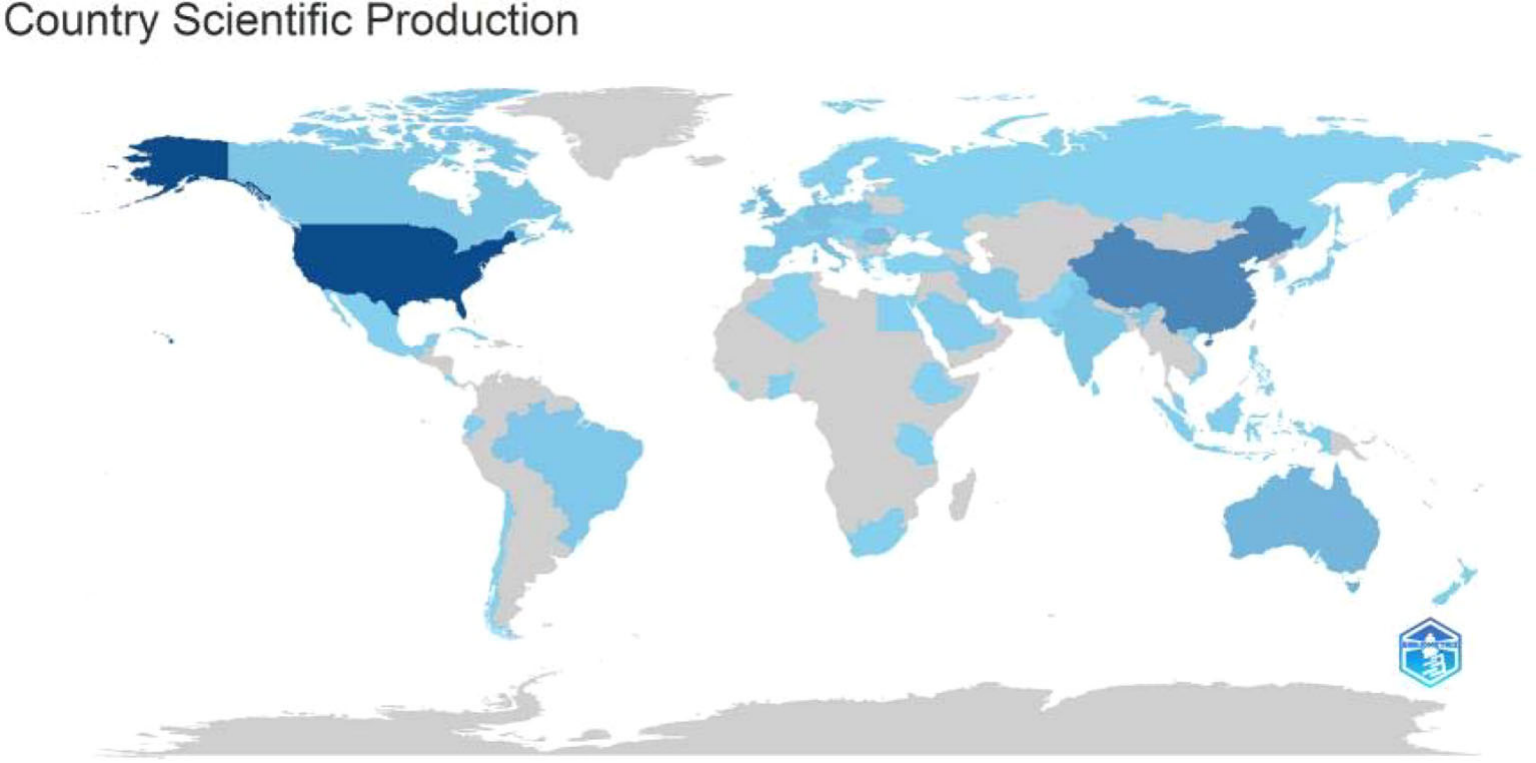
Global distribution of country scientific production. This choropleth map illustrates relative scientific output: dark blue indicates the highest levels (e.g., United States, China), light blue denotes moderate activity (e.g., Canada, European nations, Australia, Brazil), and gray signifies no data or output below threshold. The visualization highlights the concentration of research activity in North America, East Asia, and Western Europe.

A total of 68 different countries/regions participated in research on the gastrointestinal microbiota and chronic wounds (**Figure 3**). Comprehensive analysis of the top 10 countries/regions and institutions revealed that the United States ranked first as the core with 203 publications; China (86 articles), the United Kingdom (47 articles), and Australia (37 articles) formed the second echelon; Germany, Denmark, and other countries had relatively few publications (fewer than 30 each) (**Table 1**).

**Table 1:** Top ten countries/regions and institutions studying intestinal microbiota and chronic refractory wounds.

Rank	Country	Documents	Citations	Total link strength	Institutions	Documents	Citations	Total link strength
1	usa	203	10795	80	univ penn	17	1851	4383
2	england	47	2943	44	univ copenhagen	14	877	4005
3	australia	37	1970	30	southwest reg wound care ctr	12	1012	3339
4	peoples r china	86	1604	22	univ montpellier	8	326	3216
5	denmark	17	931	21	univ manchester	11	669	3110
6	germany	23	461	16	montana state univ	8	1167	2731
7	italy	21	1111	14	copenhagen univ hosp	7	154	2576
8	india	23	285	13	texas tech univ	10	218	2549
9	netherlands	14	385	13	carol davila univ med& pharm	9	295	2233
10	switzerland	11	757	13	univ calif riverside	9	306	2364

This table ranks countries/regions and institutions by total link strength, reflecting research output and collaboration intensity. The USA leads globally (203 documents, 107,995 citations, link strength 80), while the University of Pennsylvania (USA) tops institutions (17 documents, 1,851 citations, link strength 4,383). The data underscores North America, Europe, and East Asia as key contributors to this field.

In terms of academic impact, the United States had a total of 10,795 citations; the United Kingdom (2,943 citations), Australia (1,970 citations), and China (1,604 citations) also had high citation volumes. Most countries had fewer than 1,000 citations, indicating limited academic influence.

**Figure 4:**
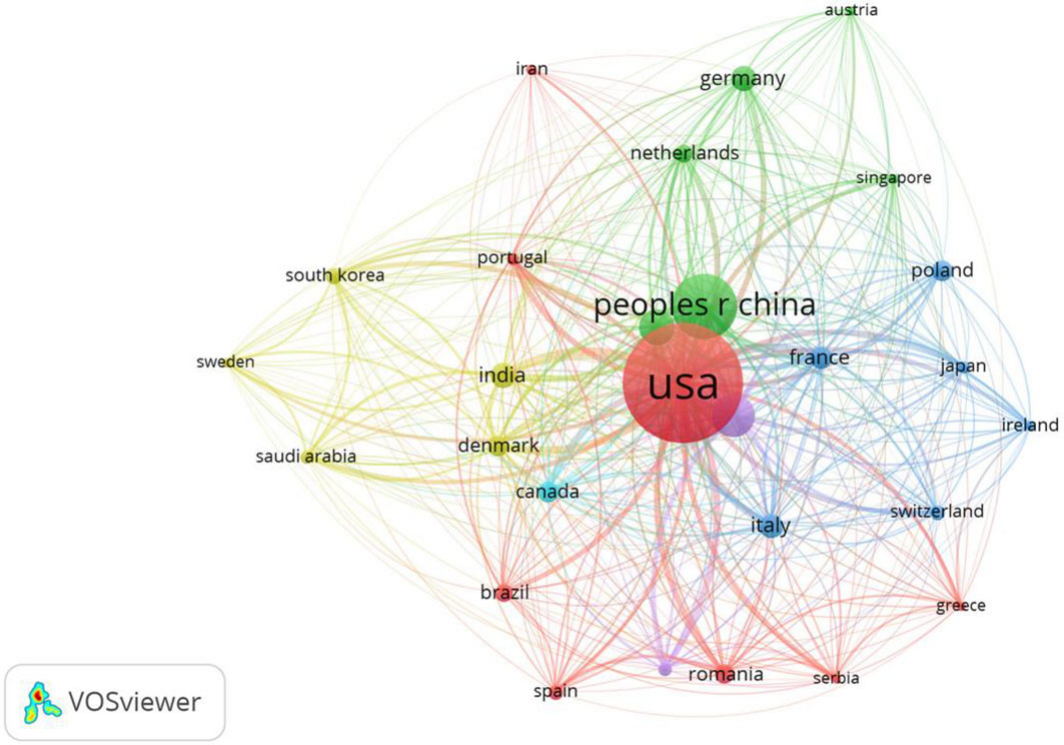
The map of cooperation between countries and regions of intestinal microbiota and chronic refractory wounds published globally. This network visualization, generated by VOSviewer, depicts international research collaboration, where node size and color intensity reflect the volume of scientific output, and line thickness indicates the strength of collaborative ties. The USA (red node) and the People’s Republic of China (green node) emerge as the most prominent contributors, with extensive cross-border links to Europe, Asia, and the Americas, underscoring their central roles in driving global research efforts in this field.

**Figure 4** shows that the United States served as the core of international cooperation with a link strength of 80; the United Kingdom (link strength = 44) and Australia (link strength = 30) had a high degree of cooperation and were key to regional collaboration. Other countries generally had a link strength of less than 25, indicating weak connectivity. Nodes of the same color represent clusters with significant cooperative relationships, and arcs represent cooperation between different countries/regions—with thicker arcs indicating closer cooperative ties. Both China and the United States maintained cooperative relationships with numerous countries, among which Sino-US cooperation was the most frequent.

**Figure 5:**
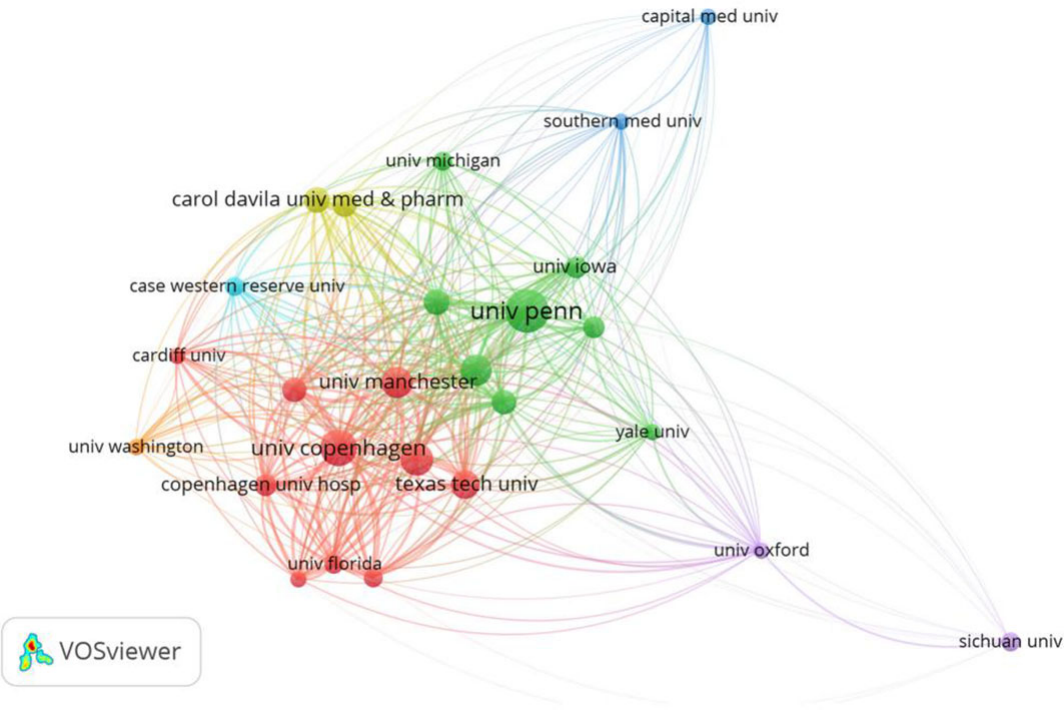
Visualization of collaborative network of research institutions involved in intestinal microbiota and chronic refractory wounds. This VOSviewer network map illustrates institutional research collaborations, where node size reflects output volume and line thickness indicates collaboration strength. The University of Pennsylvania (green node) emerges as a central hub, with extensive ties to institutions across North America, Europe, and Asia, underscoring its pivotal role in driving global research efforts in this field.

A total of 1,029 institutions participated in the research in this field. After screening with a minimum of 5 publications, 26 institutions were included in the analysis, and the top 10 institutions with the highest publication output published 105 articles (**Figure 5**). The University of Pennsylvania (Univ. Penn.) contributed 17 articles with 1,851 citations, ranking first in both publication volume and citation count. The University of Copenhagen (Univ. Copenhagen) followed with 17 articles (tied with Univ. Penn. for first place in publication volume) and 877 citations. The Wound Care Center of Southern Medical University (Southern Med. Univ. Wound Care Ctr.) ranked third with 12 articles. Notably, the most productive institutions were mainly from Europe and the United States, and all institutions listed in the table were from the USA, Denmark, the UK, and other countries.

The “Total link strength” in the table reflects the intensity of institutional collaboration: Univ. Penn. ranked first with a link strength of 4,383, indicating the most extensive collaborative network. Univ. Copenhagen (4,005) and Southern Med. Univ. Wound Care Ctr. (3,339) were core nodes in the collaboration network. Regarding collaboration characteristics, institutions with higher link strength (e.g., the University of Pennsylvania and the University of Copenhagen) displayed denser “curves” (collaborative associations), indicating collaboration with more institutions and a higher degree of cooperation (**Figure 5**).

### 3.3 Author and co-cited author

A total of 3112 authors participated in research on chronic wounds and intestinal microbiota (**Table 2**). Using the minimum number of publications as the threshold, 81 authors were included, and those with the highest publication counts were analyzed. Among these, Elizabeth A. Grice was a leading contributor, with 14 publications; Catherine Dunych-Remy, Jean-Philippe Lavigne, and Irena Pastar followed closely, each with 8 publications.

**Table 2:** Main authors studying intestinal microbiota and chronic refractory wounds.

Rank	Author	Documents	Citations	Total link strength
1	dumych-remy, catherina	8	326	14
2	leving, jean-philippe	8	326	14
3	holian, alina maria	6	196	13
4	lazar, veronica	6	284	13
5	mihai,mara madalina	5	195	13
6	popa, liliana gabriela	5	195	13
7	sotto, albert	6	267	12
8	lazarus, gerald s.	5	532	7
9	zenilman, jonathan m.	5	532	7
10	pastar, irena	8	338	6
11	tomic-canic, marjana	6	333	6
12	gardner, sue e.	6	962	5
13	grice, elizabeth a.	14	1844	5
14	stewart, philip s	5	332	5
15	phillips, caleb d.	5	412	4
16	wollott, randall d.	7	996	4
17	bjarnsholt,thomas	7	332	2
18	manale, matthew	7	292	2

This table lists the most active authors, ranked by total link strength (a measure of collaboration intensity). Dumych-Remy, Catherina and Leving, Jean-Philippe lead with 8 documents, 326 citations, and a total link strength of 14.

### 3.4 Keyword analysis

High-frequency keywords can indicate the evolving research frontiers in certain knowledge fields. Using VOSviewer, we identified 1472 author keywords, among which 50 author keywords with an occurrence frequency of at least 5 times were defined as high-frequency keywords. Analysis of the frequency and link strength of these high-frequency keywords revealed distinct research foci.

**Table 3** presents the distribution and co-occurrence characteristics of high-frequency keywords in this field. Among the basic distribution of high-frequency keywords, “wound healing” had the highest frequency (n=91) and also ranked first in link strength (150), followed by “biofilm” (n=72, link strength=130) and “chronic wound” (n=69, link strength=117). In addition, the frequencies of “bacteria”, “infection”, and “micro biota” were 19, 32, and 18, respectively. This distribution clearly reflects that the core research in this field focuses on key topics such as wound healing, biofilm formation, microbial regulation, chronic wounds, and bacterial infection, forming a research pattern with wound healing as the core and multiple related topics developing synergistically.

**Table 3:** High-frequency author keywords.

Rank	Keyword	Occurrences	Total link strength
1	wound healing	91	150
2	biofilm	72	130
3	chronic wound	69	117
4	microbiome	57	112
5	wound	31	73
6	infection	32	63
7	diabetic foot ulcer	30	54
8	inflammation	25	39
9	wound dressing	11	36
10	probiotics	15	35
11	bacteria	19	34
12	diabetes	17	34
13	microbiota	18	34
14	wound care	8	32
15	staphylococcus aureus	18	30
16	pseudomonas aeruginosa	15	29
17	skin microbiome	15	27
18	skin	12	26
19	wound infection	14	22
20	debridement	6	19
21	oxidative stress	7	18
22	antibiotic resistance	11	17
23	diabetic foot	7	17
24	antimicrobial resistance	8	15
25	dysbiosis	6	15
26	healing	5	14
27	antibiotics	6	13
28	gut microbiota	14	13
29	skin microbiota	10	13
30	ulcer	5	13
31	cancer	5	12
32	immunomodulation	5	12
33	periodontitis	6	12

This table ranks keywords by total link strength, with wound healing (150) and biofilm (130) as dominant themes, highlighting core research on microbiome, infection, and chronic wound management.

**Figure 6:**
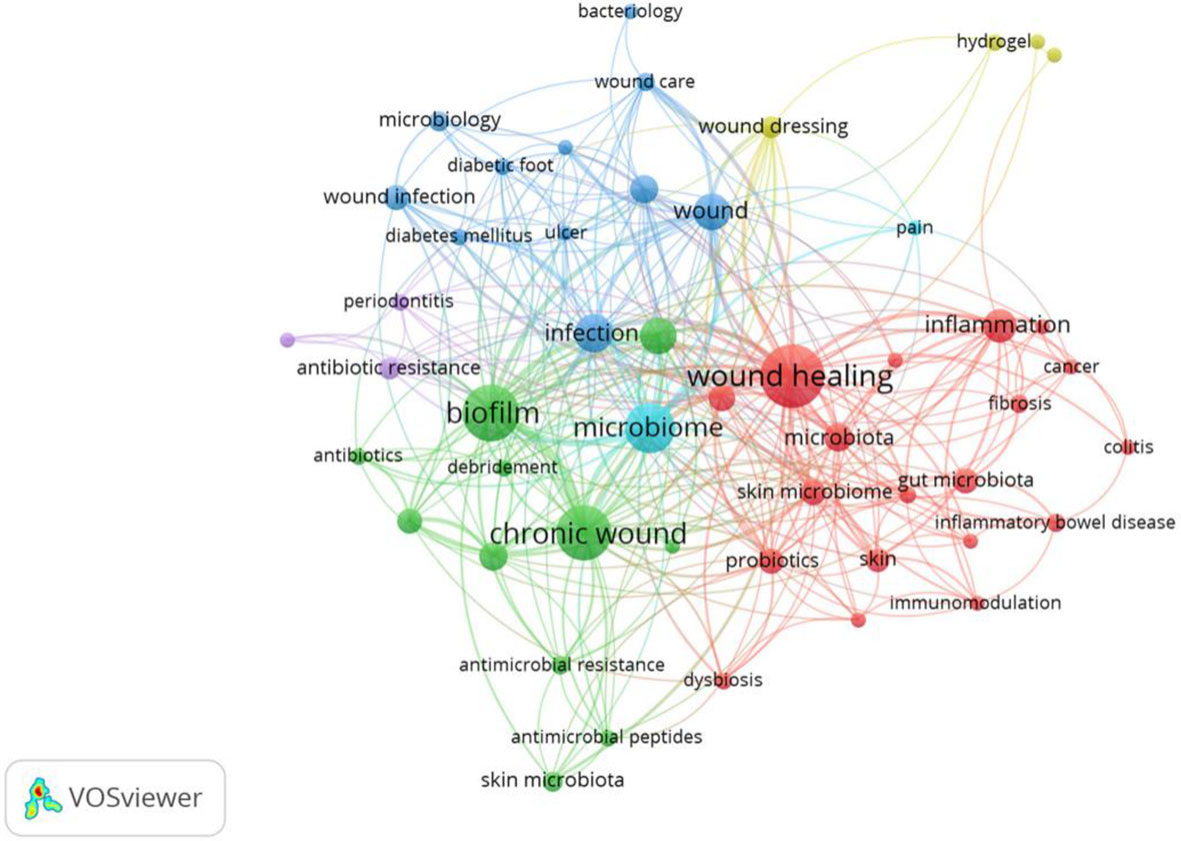
Co-occurrence network diagram of intestinal microbiota and keywords related to chronic refractory wounds. This VOSviewer network map illustrates keyword co-occurrence, where node size reflects frequency and line thickness indicates association strength. Wound healing (red node) is the central hub, closely linked to biofilm, microbiome, chronic wound, and infection, highlighting the core interplay between microbial communities and chronic wound pathophysiology.

In the keyword co-occurrence network map (**Figure 6**), the node size of the keyword co-occurrence network corresponds to its co-occurrence frequency, the link thickness represents the co-occurrence intensity, and clusters with the same color reflect the close correlation of research topics, forming several core clusters. The red cluster takes “wound healing” as the core, with a total link strength of 150, making it the core node with the most extensive correlations among all keywords. Meanwhile, it covers several key keywords including “infection” (n=39), “skin” (n=26), “skin micro biome” (n=27), “probiotics” (n=35), and “gut micro biota” (n=13). The research in this cluster focuses on the pathophysiological processes of wound healing (e.g., inflammatory response), the correlation mechanisms between skin/intestinal microflora and wound healing, and the application of microbial regulation methods such as probiotics in wound healing, forming a complete research chain of “wound healing—inflammation—microflora—probiotic intervention”, which embodies the systematic research idea from pathological mechanisms to intervention strategies in this field.

The keywords of the green cluster include “Chronological Wound” (n=117), “Biofilm” (n=130), “Diabetic Foot Ulcer” (n=54), “Staphylococcus Aureus” (n=30), and “Pseudomonas aeruginosa” (n=29). The core research of this cluster focuses on the infection characteristics of chronic wounds, focusing on the colonization and biofilm formation mechanisms of pathogenic bacteria (e.g., Staphylococcus aureus and Pseudomonas aeruginosa) in chronic wounds, as well as the antimicrobial resistance induced thereby, which provides a key research direction for the precise prevention, control, and treatment of chronic wound infections.

Cluster 3 (blue cluster, cluster=3) takes “bacteria” (n=34), “infection” (n=63), and “wound” (n=73) as the core, and is associated with “diabetic foot” (n=17), “wound infection” (n=22), and “healing” (n=14). Its research focuses on the correlation mechanism between bacteria and wound infection. Cluster 4 (yellow cluster, cluster=4) covers keywords such as “anti-infection” (n=3), “hydrogel” (n=4), and “ulcerative colitis” (n=2). The research direction of this cluster is relatively focused: on the one hand, it pays attention to the role of anti-inflammatory mechanisms in related diseases (e.g., ulcerative colitis and wound inflammation); on the other hand, it explores the application potential of materials such as hydrogel in the treatment of inflammation-related diseases. This reflects the extension of this field to anti-inflammatory treatment and biomaterial application, providing a research breakthrough for the integration of interdisciplinary technologies.

The keywords of the purple cluster (cluster=5) are “biological resistance” (n=17), “periodontitis” (n=12), “chronic infections” (n=6), etc. Its research focuses on the occurrence mechanisms and prevention/control strategies of antibiotic resistance in chronic infections. Cluster 6 is an orange cluster, with “micro biome” (n=112) and “pain” (n=10) as core keywords. The research of this cluster focuses on the correlation mechanism between microbiome imbalance and pain symptoms, which is an important branch of microbiome research extended to the correlation with clinical symptoms.

## 4 Discussion

Based on the “gut-skin axis” hypothesis, intestinal flora affects the healing process of chronic wounds by regulating systemic immunity and inflammatory response. This research mechanism has become a new research direction and potential therapeutic target in the field of wound repair (Huang et al., 2025). The gut-skin axis is a bidirectional regulatory pathway between the intestine and the skin, composed of immune, metabolic, neuroendocrine, and microbial signals (Scharschmidt et al., 2013). Dysbiosis of the gut microbiota can affect the local skin microenvironment, barrier function, and immune response through circulating metabolites, inflammatory factors, and immune cell migration, thereby delaying the repair process of chronic refractory wounds (De Pessemier et al., 2021). In recent years, numerous studies have confirmed that gut microbes and their metabolites are the core mediators of gut-skin axis in regulating wound healing, among which short-chain fatty acids (SCFAs) are considered the most critical effector molecules (Trompette et al., 2014; Cani et al., 2009).

Short-chain fatty acids (e.g., acetic acid, propionic acid, butyric acid) are produced by the fermentation of dietary fiber by beneficial gut bacteria. They can reach skin tissues through the systemic circulation and directly act on keratinocytes to enhance the structure and function of the skin barrier. SCFAs can upregulate the expression of tight junction proteins (Occludin, Claudin-1) and filaggrin (Filaggrin), promote keratinocyte differentiation and stratum corneum maturation, and improve the integrity of the skin mechanical barrier (Furusawa et al., 2013). Meanwhile, SCFAs can activate the Nrf2 antioxidant pathway, reduce local oxidative stress damage in wounds, and maintain the survival and proliferation of epithelial cells (Zhang et al., 2023). As a preferred energy substrate for epithelial cells, butyric acid can accelerate the re-epithelialization process and has a clear promoting effect on the barrier repair of refractory wounds such as diabetic foot ulcers (Chen et al., 2018).

Diabetes mellitus is a metabolic disorder characterized by persistent hyperglycemia, which not only impairs systemic immune function and microcirculation but also disrupts the homeostasis of the gut microbiota and intestinal barrier, leading to intestinal leakage (increased intestinal permeability) (Brownlee et al., 2005; Cani et al., 2007). Intestinal leakage refers to the dysfunction of the intestinal epithelial barrier, which, under normal circumstances, is a physical and functional barrier that prevents antigens, toxins, and microorganisms in the intestinal lumen from entering the systemic circulation (Turner, 2009). In diabetic patients, oxidative stress, inflammation, and changes in gut microbiota composition (dysbiosis) induced by long-term hyperglycemia can disrupt the tight junctions between intestinal epithelial cells, thereby increasing intestinal permeability (Zhang et al., 2020; Thaiss et al., 2018; Mostafavi Abdolmaleky et al., 2024).

The gut-skin axis is a bidirectional communication pathway between the gastrointestinal tract and the skin, playing a key role in mediating the impact of intestinal leakage on chronic wound healing in diabetic patients (Chai et al., 2024). Gut microbiota and their metabolites (e.g., short-chain fatty acids, lipopolysaccharides) are key mediators of this axis. In the context of diabetes and intestinal leakage, lipopolysaccharides (LPS) and other pro-inflammatory molecules transfer from the intestinal lumen to the blood, triggering a systemic low-grade inflammatory response (Ghosh et al., 2020). This systemic inflammation can further exacerbate skin inflammation, impair the proliferation and migration of keratinocytes and fibroblasts, inhibit angiogenesis, and reduce the production of growth factors—all of which are essential processes for effective wound healing.

In addition, diabetic dysbiosis and intestinal leakage can alter the balance between pro-inflammatory and anti-inflammatory cytokines in the body. Chronic refractory wounds are often accompanied by persistent excessive inflammation, characterized by the dominance of M1-type macrophages and the massive release of pro-inflammatory factors (TNF-α, IL-6, IL-1β). SCFAs can regulate the immune response through dual pathways: inhibiting HDAC and activating GPR41/43 receptors. Specifically, they promote the polarization of macrophages toward the M2 anti-inflammatory repair phenotype (Kelly et al., 2015); enhance the function of regulatory T cells (Treg) to inhibit excessive inflammation (Trompette et al., 2022); and reduce the activity of the NF-κB pathway to decrease the release of pro-inflammatory factors (Zhang et al., 2023). Through the above mechanisms, SCFAs can shift wounds from a “chronic inflammatory state” to a “repair state”, creating conditions for granulation tissue growth and angiogenesis (Kelly et al., 2015).

Gut/skin microbiota transplantation has emerged as a novel strategy to regulate the gut-skin axis and promote wound healing. Basic studies have shown that gut probiotic transplantation can increase the host’s SCFA levels, improve the wound immune microenvironment through the gut-skin axis, and accelerate wound healing in diabetic mice (Huang et al., 2025). In terms of clinical research, both local microecological intervention and fecal microbiota transplantation (FMT) in patients with chronic ulcers have been observed to improve wound healing rate, reduce inflammation, and shorten healing time (Vassallo et al., 2025; Trompette et al., 2022). A recent clinical cohort study confirmed that patients with chronic refractory wounds have significantly reduced gut microbiota diversity, increased opportunistic pathogens, and significantly decreased SCFA-producing bacteria, suggesting that targeted restoration of microbiota structure can become a new therapeutic approach (Soldevila-Boixader et al., 2025).

### 4.1 Research evolution and development trend

The analysis shows that the research output in this field has been growing steadily since 2005, especially after 2020. With the development of modern microbiology, “gut-skin axis” has been re-examined and formally put forward, emphasizing the core role of intestinal flora in regulating skin homeostasis (Arck et al., 2010). Especially the systematic review by Salem et al., established the theoretical framework of intestinal microbiota as the main regulator of “intestinal-skin axis” (Salem et al., 2018). In recent years, this concept has been further extended to the field of wound repair, revealing the specific mechanism that intestinal flora imbalance affects the healing process of chronic wounds through systemic inflammation, and has become the research frontier in this field (O'Neill et al., 2016). After 2020, keywords such as “diabetic foot ulcer” and “*in situ* detection” emerged, as well as “intestinal homeostasis” (Lynch and Pedersen, 2016; Clemente et al., 2018).With the increase of related research, the research is going deeper into more specific disease models, more advanced detection technologies and more complex interaction mechanisms between systems.

Based on keyword evolution and cluster analysis, the future research trends can be summarized into several key directions. For example, deepening the mechanism and cross-system regulation, the “skin-gut axis” depends on a complex multi-system network. Metabolites of intestinal flora are key signal molecules. For example, short-chain fatty acids can enter the systemic circulation, systematically regulate the balance between pro-inflammatory and anti-inflammatory factors by binding to receptors on immune cells, and create a systemic immune environment conducive to skin repair (Ney et al., 2023). This effect begins with the intestine itself: metabolites prevent chronic systemic inflammation caused by endotoxin translocation by maintaining the integrity of intestinal barrier, which is a prerequisite for affecting the skin microenvironment (Akhtar et al., 2022). In addition, dysbacteriosis can change the production of neurotransmitter precursors, or directly produce metabolites such as phenylacetylglutamine, which may act as endocrine messengers and affect the release of neuropeptides in hypothalamus-pituitary-adrenal axis or local skin, thus regulating skin inflammation, sensation and vascular function (Bibolar et al., 2025). Animal experiments have confirmed that mice lacking microbial flora show abnormal wound inflammatory reaction and scar formation, which reversely verifies the systematic regulation of microbial signals on skin regeneration process (Khadka et al., 2023).

Based on the in-depth understanding of the “skin-intestine axis” mechanism, developing accurate intervention strategies for intestinal microecology has become a new frontier in the treatment of chronic wounds. These strategies aim to systematically improve the inflammation and immune status of the host by targeted regulation of the composition or function of intestinal flora, thus creating a favorable systemic environment for wound healing. At present, a variety of microecological targeted therapies are being studied in clinical transformation: oral administration of specific probiotics (such as Lactobacillus and Bifidobacterium) and prebiotics through nutritional supplement therapy has been proved by many preclinical and preliminary clinical trials to have an auxiliary therapeutic effect on chronic wounds including diabetic foot ulcers by regulating immunity and enhancing barrier function (Hussain et al., 2025). However, bacterial remodeling therapy can be a more thorough intervention. fecal microbial transplantation has transplanted the whole functional bacterial ecosystem of healthy donors to patients, and has achieved curative effects in other diseases closely related to bacterial imbalance (Kelly et al., 2021). Although the clinical research in the field of wound is still in its infancy, its potential to promote tissue regeneration by systematically reshaping host-microorganism interaction has been supported by theory and animal experiments (Karimi et al., 2024).

In order to realize individualized and accurate diagnosis and treatment of chronic wounds and their systematic background (such as intestinal flora), “Multiomics technology (metagenome, metabolomics, protein group) integrated analysis” is the key method to analyze host-microorganism interaction and find healing biomarkers (Mihai et al., 2024). Metagenomics reveals microbial community composition and functional genes; Metabonomics identifies key metabolites with biological activity (such as short-chain fatty acids and inflammatory mediators); Protein omics reflects the host’s immune and repair response. This integration can dynamically describe the global map of “host-microorganism” interaction and identify specific biomarker combinations related to the healing process (Daeschlein et al., 2017), or use advanced bioinformatics tools to deeply mine massive omics data, for example, to realize strain level resolution to track pathogenic clones (Zhang et al., 2026). Or infer the functional activity of microbial community through metabolic network model, thus transforming complex data into biological insights that can guide treatment. Laboratory analysis can be combined with bedside *in-situ* detection technology (Phalak and Henson, 2019).

### 4.2 Research limitations

The bibliometric analysis was only based on the included database literature, which may have missed some relevant studies; the in-depth excavation of research content was insufficient, and the specific molecular mechanisms of “skin-gut” interaction were not deeply analyzed. In addition, the analysis was limited to 2025, and the latest explosive hotspots emerging thereafter need to be tracked in subsequent studies. Future research can expand the scope of literature inclusion, combine basic experiments with clinical research, further clarify the core mechanisms by which intestinal flora regulate the healing of chronic refractory wounds, explore new methods and targets for wound repair targeting intestinal flora, and promote the clinical transformation and high-quality development of this field.

## 5 Conclusion

This study comprehensively presented the overall research landscape of the field of intestinal flora and chronic refractory wounds from 2005 to 2025 using bibliometric methods. It systematically sorted out the research hotspots, development trends, core institutions, and collaborative networks in this field, providing objective data support and direction guidance for subsequent related research.

The research results showed that this field has rapidly developed from an early single research perspective focusing on local microbial infections to a comprehensive disciplinary direction focusing on the systemic “skin-gut” interaction mechanism and emphasizing multi-system coordinated regulation. The regulatory role of intestinal flora in the healing of chronic refractory wounds has become the core research focus in the field. Core research institutions are mainly concentrated in Europe and the United States, among which the University of Pennsylvania, the University of Copenhagen, and other institutions have become leaders and collaborative cores in this field by virtue of their high publication volume, citation count, and cooperative link strength, providing important support for technological breakthroughs and academic exchanges in the field.

The bibliometric analysis of this study clearly revealed the development context and research gaps in the field of intestinal flora and chronic refractory wounds, confirmed the key significance of the “skin-gut” axis in chronic wound repair, and provided important theoretical references and research ideas for clinically exploring new wound repair strategies and targeting intestinal flora to improve wound healing outcomes.

## Data Availability

The raw data supporting the conclusions of this article will be made available by the authors, without undue reservation.
